# Loneliness and Life Satisfaction in Older Ethnic Minority Couples in the UK: A Dyadic Analysis of UKHLS Data

**DOI:** 10.1093/geroni/igaf122.1939

**Published:** 2025-12-31

**Authors:** Isla Rippon, Kimberley Smith, Christina Victor

**Affiliations:** Brunel University of London, Uxbridge, England, United Kingdom; University of Surrey, Guildford, England, United Kingdom; Brunel University London, Guildford, England, United Kingdom

## Abstract

Loneliness is independently associated with a range of health outcomes including reduced life satisfaction. Although there is a substantial body of research about loneliness in older adults in the UK, there is a significant evidence gap reporting experiences of loneliness of older people from ethnic minorities and how this influences measures of wellbeing such as life satisfaction. Spouse/partner interpersonal relationships may result in the loneliness experienced by one member of the couple influencing their partner’s life satisfaction as well as their own. We investigate self and partner experiences of loneliness on life satisfaction in older couples from ethnic minority communities in the UK. We focus upon the experiences of loneliness for 611 heterosexual couples from ethnic minority communities from wave 13 (2021-23) of the UK Household Longitudinal Study (UKHLS). Loneliness was measured using the 3-item UCLA scale and life satisfaction using a study specific item where higher scores indicate greater satisfaction with life. 58% of participants identified as Asian and around a third of couples were of mixed ethnicity. 24% of males and 29% of females were lonely (UCLA score of 6+) and 46% had high life satisfaction (mostly or completely satisfied with their life). Data were analysed using the Actor-Partner Interdependence Model framework. Loneliness was associated with poorer life satisfaction for both male and female partners. Male partners loneliness affected the life satisfaction of their partner. Interventions to combat loneliness in men may benefit both themselves and their partners.

